# Reply to the comment by Osuka et al.

**DOI:** 10.1186/s40560-020-00463-2

**Published:** 2020-07-08

**Authors:** Akira Endo, Atsushi Shiraishi, Kiyohide Fushimi, Yasuhiro Otomo

**Affiliations:** 1 Trauma and Acute Critical Care Medical Center, Tokyo Medical and Dental University Hospital of Medicine, 1-5-45 Yushima, Bunkyo-ku, Tokyo, 113-8510 Japan; 2 Emergency and Trauma Center, Kameda Medical Center, 929 Higashicho, Kamogawa, Chiba, Japan; 3 Department of Health Policy and Informatics, Tokyo Medical and Dental University Graduate School of Medicine, 1-5-45 Yushima, Bunkyo-ku, Tokyo, Japan

## Abstract

Patient transfer between hospitals can be one of the biases when evaluating the hospital performance in severe burn care. Optimal handling of such a population is challenging in the analysis of an inpatient database not specialized for burn due to the lack of detailed information.

We would like to thank Osuka et al. for their comments regarding our recent article [1], in which volume-outcome relationship was assessed in patients with severe burns. In our study, we excluded patients who were transferred to other hospitals within 3 days of admission; these patients were not accounted for in the estimation of annual hospital patient volume, as well as the outcome measurement. A majority of such patients were considered as transferred to specialized hospitals for definitive care, since treatment of patients with severe burns cannot be completed in 3 days. When these patients are included in the analysis, the performance of non-specialized hospitals would be overestimated, resulting in a substantial bias.

Since this was a retrospective study analyzing a database that is not specialized for burn care, risk adjustment of patients was bound to be insufficient. To overcome this limitation, we performed several sensitivity analyses. In the analysis wherein the treatment intensity within 2 days of admission was used for the risk adjustment model (Figure S7 of the original paper [1]), considering the immortal time bias, patients who died within 2 days of admission were excluded from the analysis. In this analysis, Osuka et al. noted the apparent increased risk of mortality among low-volume hospitals compared to the analysis of the overall population (Fig. 2 of the original paper [1]) and pointed out the impact of patients who were transferred to other hospitals within 2 days of admission. However, as mentioned above, such a population was excluded from both the analyses; therefore, the results would not be affected by such patients. Furthermore, because these two analyses employed different risk adjustment models, they should not be compared directly. Patients who died within 2 days of admission might not be salvageable by any treatment, regardless of whether they were transferred from another hospital. Figure S7 of the original paper [1] suggested that even when such patients were excluded, the survival benefit was not observed in the high-volume centers. However, as Osuka et al. mentioned, some severe burn patients are transferred to well-experienced hospitals after 3 days of admission for the treatment of late-phase complications such as burn-induced sepsis. Although we made an effort to reduce the bias related to patient transfer, this subpopulation could affect our results, and it should have been mentioned as a limitation. Furthermore, the issue of overestimation of the burned area by physicians who are not familiar with burn sizing, which was noted by Osuka et al., was also the limitation in the study.

In the letter, Osuka et al. have suggested performing additional sensitivity analysis in which (1) patients who were transferred from other hospitals, (2) patients who were transferred to other hospitals, and (3) patients who died within 2 days of admission are excluded. When these criteria were applied, 2446 patients (46.5% of overall study population) from 880 hospitals were eligible for analysis. The number of the hospitals treating more than 5 patients annually was only 55 (6.3%). The plot of the generalized additive mixed-effect model in this cohort is shown in Fig. 1. The area of the standard error was very broad among hospitals treating approximately more than 5 patients annually, reflecting the reduced number of patients, which prevented us to evaluate the volume-outcome relationship in these hospitals. However, at least, the trend of the volume-outcome relationship among hospitals treating 0 to 5 patients annually was similar to the results of the original analyses. In patients with severe burn, it is common that they cannot be discharged to home directly because of compromised activity of daily living caused by long-term bed rest and hospitalization, even though the patients can be well managed and may have recovered. Furthermore, some high-volume hospitals have an increase in the number of patients by transfer from other nearby hospitals. Considering these reasons, analysis excluding patients who were transferred from or transferred to other hospitals would result in substantial selection biases.

**Fig. 1:**
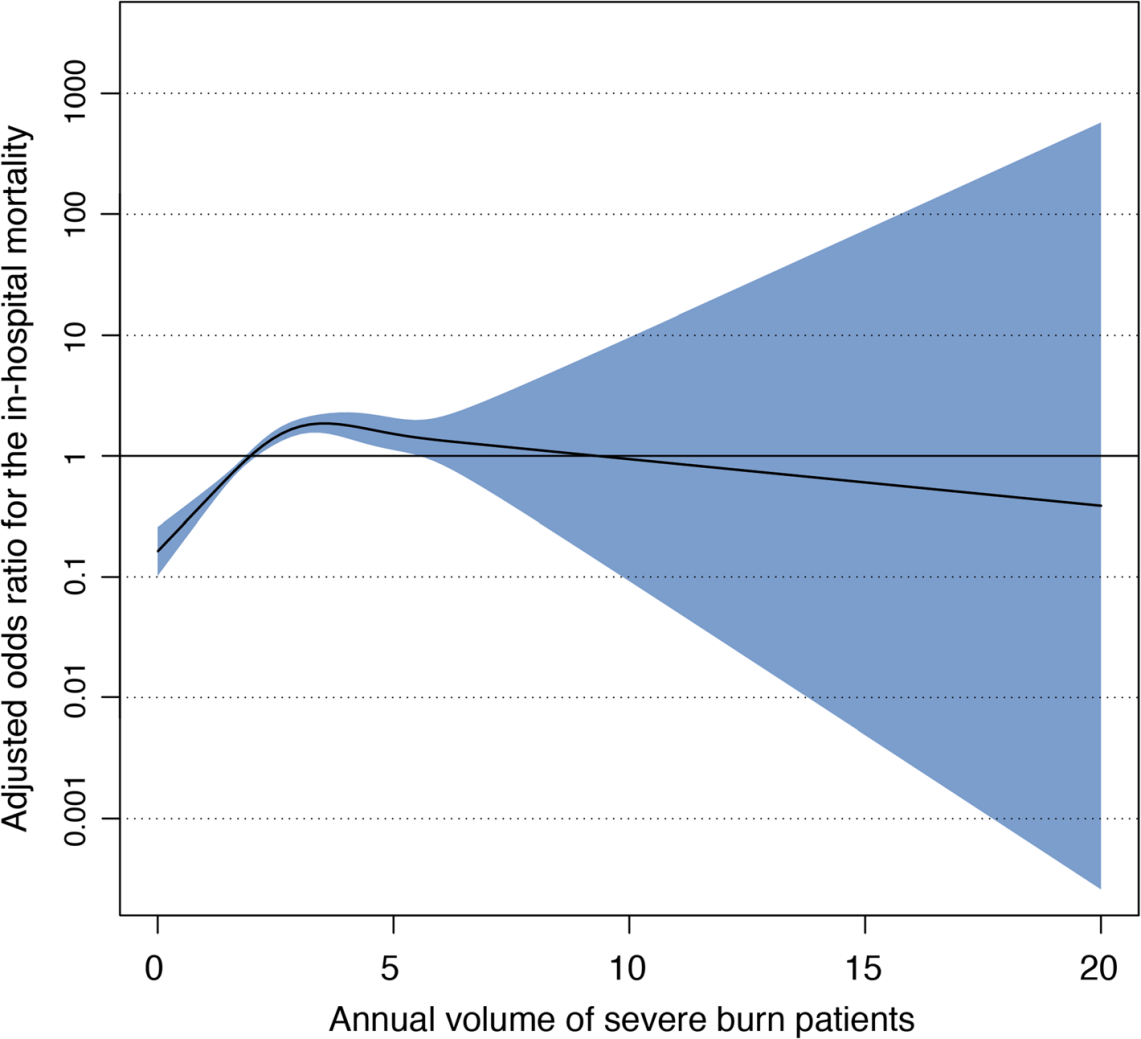
Association between annual severe burn patient volume and adjusted risk of in-hospital survival among patients who were not transferred from or transferred to other hospitals and who survived over 2 days of admission. The shaded region represents the standard errors for the point estimates. Patient severity was adjusted by prognostic burn index as a fixed effect variable. The hospital unique identifier was also adjusted as a random effect variable

Japan lacks a centralized system for severe burn patients and the number of patients in each hospital is limited. Although this study categorized hospitals based on ≤ 5 or > 5 patients annually, the number is not large enough to be a high-volume center from a global perspective. As proposed in the article, further analysis of another database, which includes information on hospitals treating a sufficient number of burn patients, is needed to validate our results.

## Data Availability

Not applicable
